# Primary ovarian high-grade endometrial stromal sarcoma: a case report

**DOI:** 10.1186/s13256-021-02986-0

**Published:** 2021-08-03

**Authors:** Ji Sun Lee, Dayong Lee, Jisun Lee, Man-Hoon Han, Dae Gy Hong, Hyun Jung Lee

**Affiliations:** 1 Department of Obstetrics and Gynecology, School of Medicine, Kyungpook National University, Daegu, 41944 Republic of Korea; 2 Department of Pathology, School of Medicine, Kyungpook National University, Daegu, 41944 Republic of Korea; 3 Department of Obstetrics and Gynecology, School of Medicine, Kyungpook National University, Kyungpook National University Chilgok Hospital, Daegu, 41404 Republic of Korea

**Keywords:** Primary ovarian high-grade endometrial stromal sarcoma, HIPEC

## Abstract

**Background:**

Primary ovarian high-grade endometrial stromal sarcoma is a very rare disease. Even though it has poor prognosis, the gold standard treatment has not been established owing to its rarity. This report aimed to present therapeutic options for primary ovarian high-grade endometrial stromal sarcoma.

**Case presentation:**

A 49-year-old Asian woman presented with disseminated intravascular coagulation due to ruptured primary high-grade ovarian endometrial stromal sarcoma with multiple intraperitoneal metastases. After the initial surgery, the patient underwent adjuvant chemotherapy with three courses of Adriamycin (75 mg/m^2^). We performed the secondary debulking operation including total hysterectomy, metastasectomy, omentectomy, peritonectomy, appendectomy, and hyperthermic intraperitoneal chemotherapy (paclitaxel 175 mg/m^2^). Currently she has been alive for 28 months under a new chemotherapy regimen.

**Conclusion:**

We suggest cytoreductive surgery with hyperthermic intraperitoneal chemotherapy could be a therapeutic option for primary high-grade ovarian endometrial stromal sarcoma with peritoneal dissemination.

## Background

Endometrial stromal sarcoma (ESS) is a rare malignant tumor of the uterus, which accounts for 0.2% of all uterine malignancies and less than 10% of the all uterine sarcomas [1, 2]. ESS originates from invasive proliferation of cells that resembles normal proliferative endometrial stromal cells [3]. The mesenchymal neoplasm occurs mainly in the uterine corpus, but it also originates from extrauterine sites such as ovary, vagina, Fallopian tube, bladder, small bowel, colon, peritoneum, etc. [4, 5]. Although uterine endometrial stromal sarcoma is a relatively well-known disease, data regarding the management of primary ovarian ESS are scarce and limited due to its rarity. To date, reported cases of ovarian ESS are less than 100, and most of them are low-grade ESSs [6].

While low-grade ESS shows an indolent clinical course and a relatively good prognosis, high-grade ESS has poor prognosis due to rapid progression of the disease and high incidence of recurrence and metastasis. Treatment includes surgery and adjuvant therapies including chemotherapy and radiation therapy, but the gold standard has not been determined because of the limited number of cases [5, 7].

Here we describe a case of ruptured primary high-grade ovarian ESS with multiple intraperitoneal metastases being treated with cytoreductive debulking surgery and hyperthermic intraperitoneal chemotherapy (HIPEC) followed by adjuvant chemotherapy.

## Case presentation

**Fig. 1.**
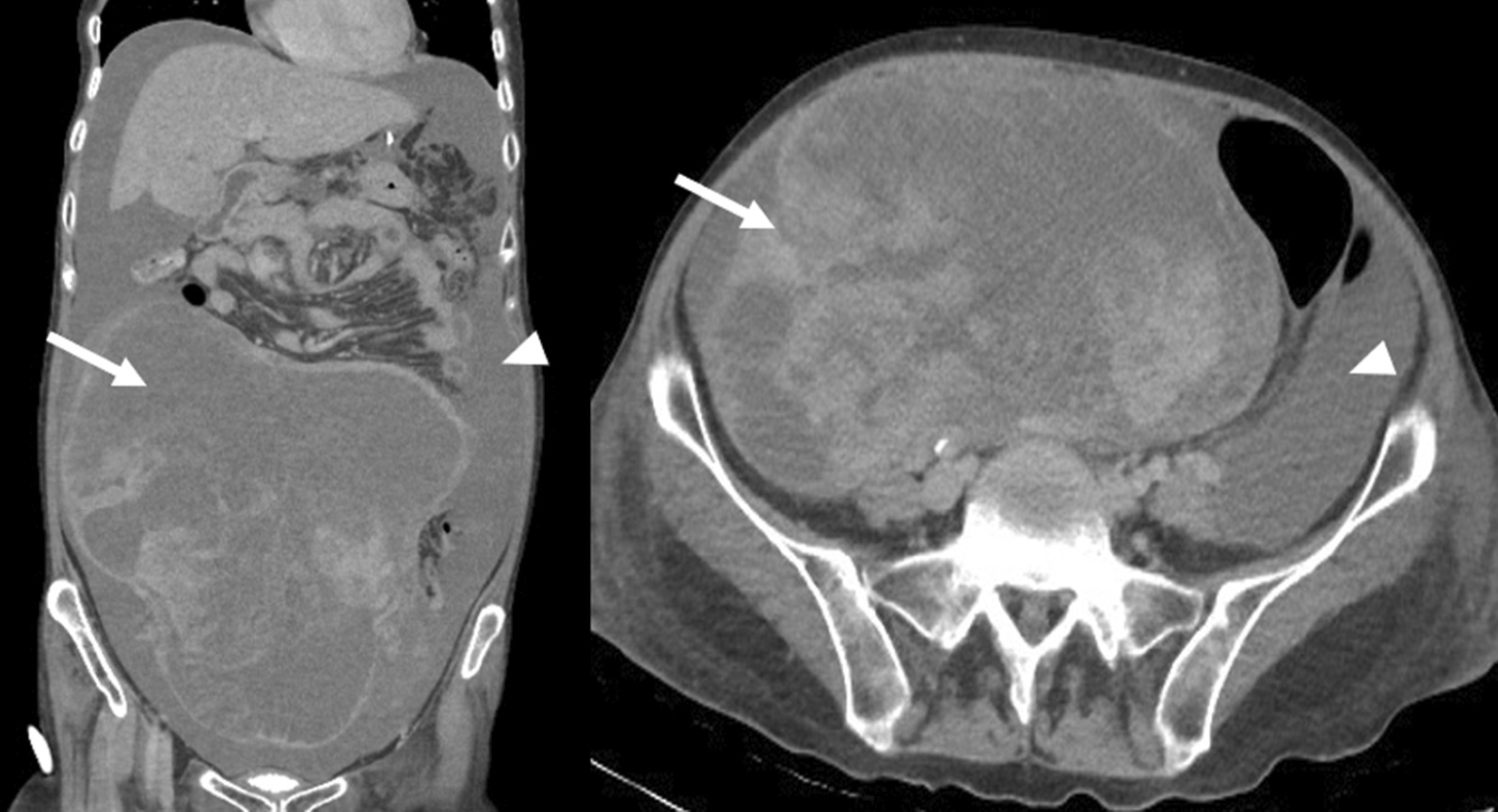
Computed tomography scan showed a 30-cm-sized irregularly hyperenhancing mass (arrow) with large amount of ascites (arrow head) and peritoneal thickening suggesting ovarian malignancy with peritoneal carcinomatosis

A 49-year-old Asian woman presented with severe abdominal distension and dyspnea. She suffered from the feeling of swelling abdomen, dull nature abdominal discomfort and pain, edema in both legs, and dyspnea from 3 weeks before her visit. She visited another hospital 2 days prior to her visit and was transferred to our emergency room owing to huge ovarian cystic mass occupying almost all of the abdominal cavity and large amount of left pleural effusion on computed tomography (CT) scan. On initial vital sign assessment, her blood pressure was stable and she had tachycardia but no fever. Her laboratory findings showed anemia (hemoglobin 8.3 g/dL), leukocytosis [white blood cells (WBC) 21.00 × 10^3^/μL], C-reactive protein (CRP) elevation of 25.8 mg/dL with prolongation of prothrombin time (PT) (90.4 seconds), PT international normalized ratio (INR) [8], and activated partial thromboplastin time (aPTT) (no coagulation), which could possibly imply disseminated intravascular coagulation (DIC). Physical examination showed abdominal distension, severe tenderness, and rebound tenderness in the whole abdomen. Due to severe dyspnea and abdominal distension, chest arrow insertion at left lung and paracentesis were performed, draining approximately 850 mL and 2 L, respectively. While performing transfusion, we reexamined dynamic abdomen-pelvis CT scan and found 30-cm-sized multiseptated cystic mass and peritoneal thickening with large amount of ascites suggesting ovarian malignancy and peritoneal carcinomatosis (Fig. 1). The patient’s preoperative cancer antigen 125 (CA125) level showed a significant increase with 674.3 U/mL, human epididymis protein 4 (HE4) was 286.4 pmol/L, and premenopausal/postmenopausal risk of ovarian malignancy algorithm (ROMA) index was 86.67%/92.83%. The other tumor markers such as carbohydrate antigen 19-9 (CA19-9), carcinoembryonic antigen (CEA), and alpha-fetoprotein (AFP) were within normal range. With all the results combined, rupture of a malignant ovarian tumor was suspected, and an emergent operation was planned.

**Fig. 2.**
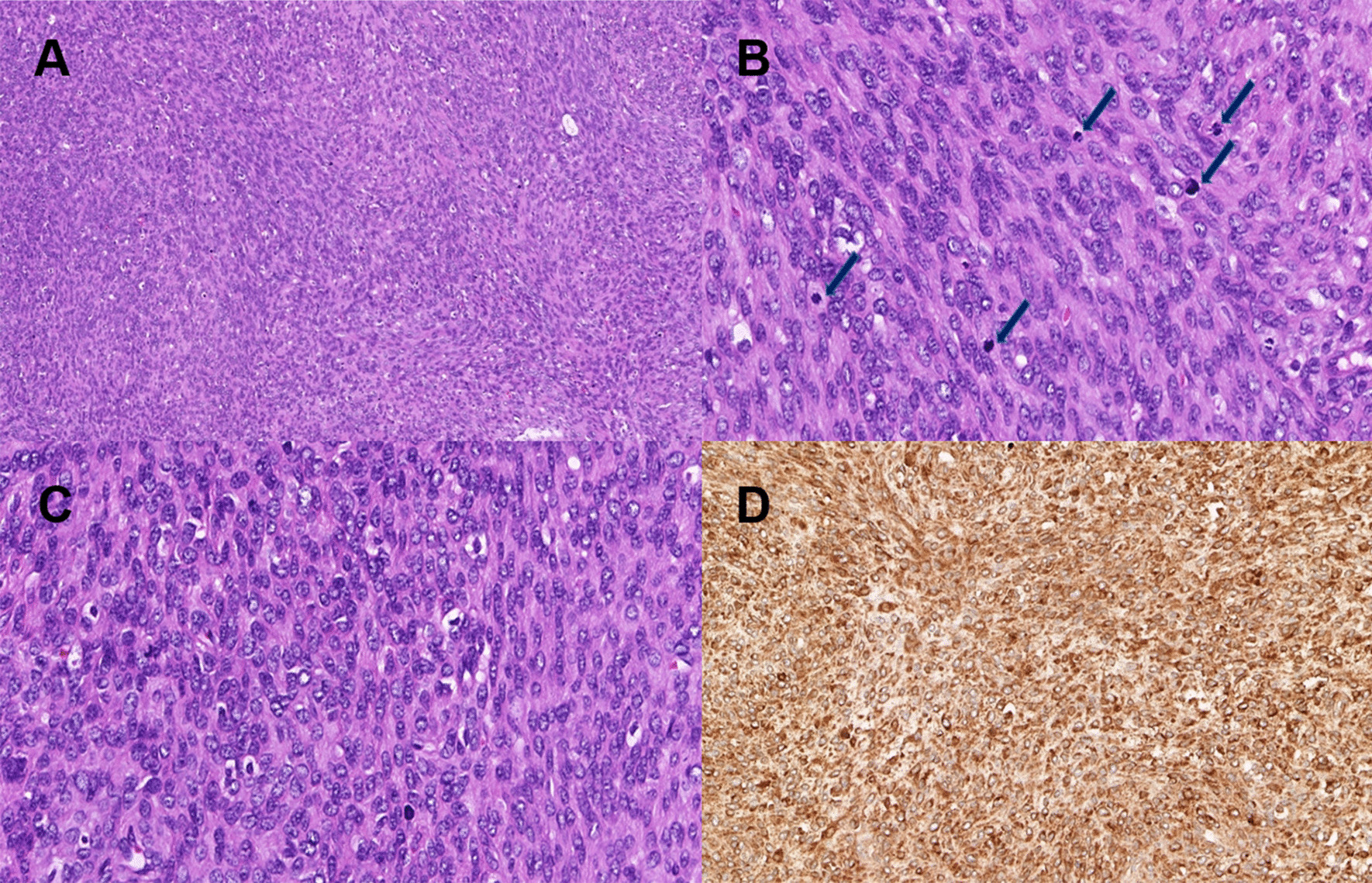
The tumor consisted of monotonous uniform cells with endometrial stromal differentiation (**A**). Mitotic figures (arrows) were frequent (**B**), and highly atypical neoplastic cells were noted (**C**). On immunohistochemistry, tumor cells were positive for CD10 (**D**)

On the initial operation findings, ruptured 30-cm-sized right ovarian multiseptated cystic mass showing diffuse adhesion to retroperitoneum, abdominal wall, and uterus was identified. Although frozen section biopsy from the right ovarian mass was revealed as a poorly differentiated carcinoma, debulking surgery including hysterectomy could not be performed owing to persistent oozing pattern bleeding from multiple sites, which was due to DIC. We performed bilateral salpingo-oophorectomy and partial omentectomy. The pathologic diagnosis of the right ovary was high-grade stromal sarcoma with features of brisk mitosis (30–40/high-power field), focal necrosis, adenofibromatous component, endometriosis with mucinous metaplasia, and hypercellular stroma (Fig. 2). The tumor consisted of monotonous uniform cells with endometrial stromal differentiation [8] (Fig. 2A). Mitotic figures were frequent (Fig. 2B), and highly atypical neoplastic cells were noted (Fig. 2C). Metastasis to omentum was also identified. On immunohistochemistry, tumor cells were positive for CD10 (Fig. 2D), Cyclin D1, and FOXL 2 and positive focally for desmin and smooth muscle actin (SMA). They were negative for beta-catenin and inhibin A. After the surgery, endometrial biopsy was done for excluding metastasis from the endometrium, and the pathologic result was nonspecific. The patient underwent adjuvant chemotherapy with three courses of Adriamycin (75 mg/m^2^). On the follow-up CT scan that was performed 6 months after the chemotherapy, new 11-mm-sized probable seeding nodules in the right omentum and left paracolic gutter were seen. A 1.5-cm-sized partly solid nodule in the right upper lung field was also identified on the chest CT. As for the lung lesion, we had both primary and metastatic lesion in mind, and planned to perform the surgery separately.

We performed the secondary debulking operation including total hysterectomy, metastasectomy, omentectomy, peritonectomy, appendectomy, and HIPEC (paclitaxel 175 mg/m^2^). In the final pathologic report, it was confirmed that the uterine cervix, myometrium, and endometrium had no specific finding except atrophy, which excludes the possibility of endometrial origin malignancy. Peritoneum, omentum, and appendix specimens were confirmed as metastatic high-grade endometrial stromal sarcoma. Two months after the surgery, the patient underwent another right upper lung lobectomy operation, and the biopsy was revealed as adenocarcinoma, which means double primary malignancy. Currently, she has been alive for 28 months under a new chemotherapy regimen: paclitaxel (175 mg/m^2^) and ifosfamide (1.6 g/m^2^).

## Discussion

Primary ovarian endometrial stromal sarcoma is an extremely uncommon entity with only about 100 cases reported in the literature so far. The sites of extrauterine ESS include the ovaries, Fallopian tubes, broad ligament, vagina, bladder, small bowel, colon, pelvic peritoneum, mesentery, and liver, and ovary is the most common primary site of extrauterine ESS [4, 8]. Since the majority of ESS originates from uterine corpus, to prove primary ovarian ESS, a thorough pathologic evaluation of uterus is essential to exclude the possibility of metastasis, which was confirmed in our case [6]. Most of the tumors occur in postmenopausal women, especially in the fifth and sixth decade. Although patients with uterine ESS typically complain about abnormal vaginal bleeding or pelvic pain, ovarian ESS usually presents with nonspecific symptoms such as abdominal distension and pain or both and is sometimes asymptomatic. Most ovarian ESSs are diagnosed in advanced stages with tumor extension beyond ovaries [9]. It has been reported that tumor markers such as CA125 could be less sensitive in these tumors than in epithelial ovarian cancers [10], but preoperative CA125 in this case was highly elevated (674.3 U/mL) and decreased dramatically after surgery and chemotherapy to normal reference range.

Previously, ESS was categorized in low- and high-grade tumors on the basis of its mitotic counts. However, the newly released 2014 WHO classification divides “endometrial stromal and related tumors” into the following five types of tumors: endometrial stromal nodule (ESN), low-grade endometrial stromal sarcoma (LG-ESS), high-grade endometrial stromal sarcoma (HG-ESS), undifferentiated uterine sarcoma (UUS), and uterine tumor resembling ovarian sex cord tumor (UTROSCT) [11]. Regarding primary ovarian ESS, according to the 2014 WHO classification system, it can be subcategorized as low-grade ESS and high-grade ESS [6]. High-grade ESS of ovary is much rarer than LG-ESS of ovary and has a poor prognosis due to its high metastasis and recurrence rate. Our patient with huge ovarian mass over 30 cm also presented with multiple metastases—disease extension to peritoneum, mesentery, appendix—at the time of diagnosis [International Federation of Gynecology and Obstetrics (FIGO) stage IIIC]. The pleural effusion cytology was negative.

The standard treatment of ESS confined to the uterus is total extrafascial hysterectomy with or without bilateral salpingo-oophorectomy, but the treatment of extrauterine ESS is controversial.[12] One retrospective study in 2007 showed that optimal cytoreduction to < 2 cm of the residual disease resulted in more favorable outcome in median overall survival compared with suboptimal residual disease in patients with HG-ESS (52 versus 2 months, *p* = 0.007) [5]. Systemic pelvic and paraaortic lymphadenectomy is not routinely recommended, but dissection of enlarged lymph node is indicated in the case of disseminated or recurrent disease. In cases of primary ovarian ESS, in the absence of specific data, it seems reasonable to adapt the rationale of surgical treatment of uterine sarcoma. If the lesion is confined to the ovary, total hysterectomy and bilateral salpingo-oophorectomy is the mainstay of treatment. Patients with advanced diseases need tumor debulking surgery, while the role of routine systemic lymphadenectomy is still elusive. The role of postoperative adjuvant therapies such as chemotherapy and radiation therapy is unclear and does not have specific evidence of survival benefit. [13]

There have been several retrospective reviews about the possibility of cytoreductive surgery followed by hyperthermic intraperitoneal chemotherapy (HIPEC) to be a promising treatment modality in patients with high-grade uterine sarcoma with peritoneal dissemination [14-16]. Multiple studies have already suggested survival benefits in patients with epithelial ovarian malignancies with peritoneal dissemination when treated by cytoreductive surgery with HIPEC. In addition, in peritoneal carcinomatosis of nongynecological origin such as colon or in primary peritoneal malignancies, survival benefit of cytoreductive surgery and HIPEC has been demonstrated in a number of studies [17, 18].

## Conclusion

Primary ovarian high-grade endometrial stromal sarcoma (ESS) is a very rare disease. Even though it has poor prognosis, the gold standard treatment has not been established owing to its rarity. This report aimed to present therapeutic options for primary ovarian high-grade ESS. We suggest cytoreductive surgery with HIPEC could be a therapeutic option for high-grade uterine sarcomas with peritoneal sarcomatosis as well as for primary high-grade ovarian ESS with peritoneal dissemination. Although large studies might be challenging owing to the rarity of the disease, further study is needed.

## Data Availability

All data generated or analyzed during this study are included in this published article.
